# How Is the Norepinephrine System Involved in the Antiepileptic Effects of Vagus Nerve Stimulation?

**DOI:** 10.3389/fnins.2021.790943

**Published:** 2021-12-02

**Authors:** Alexandre Berger, Simone Vespa, Laurence Dricot, Manon Dumoulin, Evelina Iachim, Pascal Doguet, Gilles Vandewalle, Riëm El Tahry

**Affiliations:** ^1^Institute of Neuroscience, Université catholique de Louvain, Brussels, Belgium; ^2^Synergia Medical SA, Mont-Saint-Guibert, Belgium; ^3^GIGA-Cyclotron Research Center-In Vivo Imaging, University of Liège, Liège, Belgium; ^4^Department of Pediatric Neurology, Carol Davila University of Medicine and Pharmacy, Bucharest, Romania; ^5^Center for Refractory Epilepsy, Department of Neurology, Cliniques Universitaires Saint-Luc, Brussels, Belgium

**Keywords:** VNS, epilepsy, norepinephrine, locus coeruleus, vagus nerve, biomarker

## Abstract

Vagus Nerve Stimulation (VNS) is an adjunctive treatment for patients suffering from inoperable drug-resistant epilepsy. Although a complete understanding of the mediators involved in the antiepileptic effects of VNS and their complex interactions is lacking, VNS is known to trigger the release of neurotransmitters that have seizure-suppressing effects. In particular, norepinephrine (NE) is a neurotransmitter that has been associated with the clinical effects of VNS by preventing seizure development and by inducing long-term plastic changes that could restore a normal function of the brain circuitry. However, the biological requisites to become responder to VNS are still unknown. In this review, we report evidence of the critical involvement of NE in the antiepileptic effects of VNS in rodents and humans. Moreover, we emphasize the hypothesis that the functional integrity of the noradrenergic system could be a determining factor to obtain clinical benefits from the therapy. Finally, encouraging avenues of research involving NE in VNS treatment are discussed. These could lead to the personalization of the stimulation parameters to maximize the antiepileptic effects and potentially improve the response rate to the therapy.

## Introduction

Epilepsy is a common neurological condition that affects approximately 50 million people around the world (Scharfman, 2015). This disease is associated with high economic costs and several often-debilitating comorbidities. The main comorbidity associated with epilepsy is depression (Kogeorgos et al., 1982; Mendez et al., 1986). Epilepsy is considered to be “refractory” (or more precisely, drug-resistant) whenever a patient is not rendered seizure free with the use of at least two antiepileptic drugs at correct dosages. Approximately 30% of epileptic patients will develop a drug-resistant epilepsy (Spencer and Huh, 2008). While some individuals with refractory epilepsy can become seizure-free with a surgical resection of the epileptogenic focus, other patients will not be candidates for a surgical solution because of the presence of multifocal or generalized epilepsy or whenever the epileptogenic focus lies in eloquent cortex that cannot be removed (Englot et al., 2013).

Vagus Nerve Stimulation (VNS) has been proposed for over 30 years as an adjunctive treatment for patients with inoperable drug-resistant epilepsy. This therapy is approved in the United States for treatment-resistant depression, and also showed positive effects in multiple other medical conditions, including chronic pain, Parkinson’s disease, essential tremors, gastroparesis, chronic tinnitus, stroke, post-traumatic stress disorder, eating disorders (e.g., bulimia nervosa and morbid obesity), multiple sclerosis, migraine, and Alzheimer’s disease (Krahl et al., 2004; Beekwilder and Beems, 2010; Hays et al., 2013; Broncel et al., 2020). VNS consists of a device implanted in the upper left thoracic region with a helical electrode placed around the left cervical nerve, which delivers intermittent electrical impulses to activate the vagus nerve. It was shown that 24–48 months after the implantation of the device, 63% of patients were considered as responders (i.e., ≥50% reduction in seizure frequency) and 8.2% of implanted patients became seizure free (Englot et al., 2016). Although not totally elucidated yet, the mechanisms of action of VNS have been extensively studied. Due to the established connections of the vagal afferent pathway with structures relaying norepinephrine (NE) in the brain (Ruffoli et al., 2011), and to the role of NE in preventing or attenuating seizures (Giorgi et al., 2003; Chachua et al., 2010), the noradrenergic system has been considered as a leading player in the mechanisms of action of VNS.

In this review, after a brief overview of the functional anatomy of vagal stimulation, evidence of the critical involvement of NE in the antiepileptic effects of VNS in animal models and humans are presented and discussed. Moreover, hypotheses are formulated on how inter-individual differences in the noradrenergic system could explain differential responses to VNS treatment. Finally, future perspectives involving NE in VNS are discussed and potential avenues for research on optimization of the treatment with respect to personalized stimulation strategies are proposed.

### Functional Anatomy of Vagal Stimulation

Anatomically, the vagus nerve is the tenth of the cranial nerves and has a mixed composition. It comprises approximately 3/4 of afferent fibers carrying taste, visceral and somatic information to the central nervous system, and 1/4 of efferent fibers, which mainly conduct parasympathetic information to the heart, lungs, gastrointestinal tract and other intra-abdominal organs, but also provide motor innervation to laryngeal muscles (Aalbers et al., 2011; Bonaz et al., 2017). The composition of the vagus nerve varies across species: it was suggested that the composition of the vagus nerve of pigs is closer to humans than in comparison to rats (Vuckovic et al., 2008; Yoo et al., 2013; Stakenborg et al., 2020). In contrast, although the distribution of fibers caliber varies (with a higher percentage of medium-diameter fibers in humans compared to mice and pigs), it was shown that the overall percentage of myelinated fibers in the cervical vagus nerve is comparable between mice, pigs and humans. At the functional level, it is also worth noting that while fibers with a higher degree of myelinization are activated with a lower electrical current intensity (Groves and Brown, 2005), other factors can influence the excitation threshold of the fibers and contribute to interspecies differences. For instance, the fibrous tissues surrounding the vagus nerve are known to increase the current needed to reach the excitation threshold, as well as the electrode design, or the position of the implanted electrode used for VNS (Stakenborg et al., 2020). Therefore, the translation of results between animal and humans is possible but should be done cautiously.

Three types of vagal fibers can be distinguished based on their functional properties: A-fibers (myelinated somatic, both efferent – Aα-, and afferent – Aβ- and Aδ- somatic fibers), B-fibers (myelinated, mostly efferent visceral), and C-fibers [unmyelinated, afferent visceral and nociceptive fibers but also preganglionic parasympathetic axons arising from the dorsal motor nucleus and mainly targeting the enteric nervous system (McCorry, 2007)], which differ in diameter and electrical conductance (Terui and Koizumi, 1984). While a study hypothesized that maximal electrical stimulation of C-fibers could reduce seizures (Woodbury and Woodbury, 1990), further studies showed that the destruction of C-fibers with capsaicin did not prevent seizure suppression (Krahl et al., 2001). Moreover, although efforts have been done to characterize fiber activation as a function of different VNS parameters (McAllen et al., 2018; Chang et al., 2020; Smets et al., 2021), it was suggested that clinically applied VNS parameters do not reach the activation threshold of C-fibers (Koo et al., 2001). Altogether, these results suggest that only A and B-fibers are effectively recruited during VNS with clinical parameters.

The nucleus of the solitary tract (NTS), situated in the dorsomedial medulla, is the major sensory nucleus receiving afferences from the vagus nerve (Ruffoli et al., 2011). The NTS has wide functional efferent projections in the brain: (i) within the brainstem, toward the rostral ventromedial medulla, parabrachial nucleus, raphe nuclei and locus coeruleus (LC); (ii) toward ascending structures, such as the amygdala, cerebellum, hypothalamus, and thalamus (Fornai et al., 2011).

### The Locus Coeruleus: A Key Relay at the Entry of the Brain

Although the LC is a small, lateralized nucleus of ∼3 mm diameter with only 15.000–20.000 neurons per side in humans, the LC is, by far, the main source of NE in the brain (Aston-Jones et al., 1986; Keren et al., 2009). It is characterized by widely diffused projections to both subcortical and cortical structures. The projections of the LC are small unmyelinated fibers, forming a wide antero-posterior branching network to reach the raphe nuclei, the cerebellum, and almost all areas of the midbrain and forebrain regions with very few exceptions (hypothalamus and striatum) (Loughlin et al., 1982). NE released by the LC may exert different effects, mostly depending on the type of receptors that are present on its target neurons (Berridge and Waterhouse, 2003). However, direct excitation or inhibition does not appear to be the salient characteristics of this noradrenergic pathway: the main effects of the LC-dependent NE appear through the modulation of other inputs to target neurons (Waterhouse and Woodward, 1980).

**FIGURE 1:**
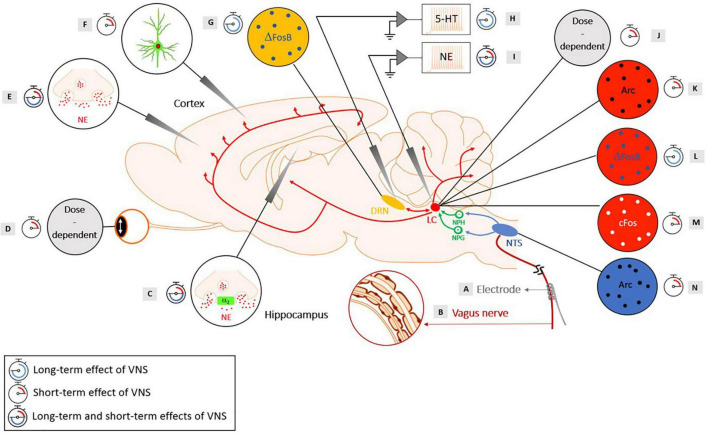
Evidence of the activation of the noradrenergic system in rats with short-term and long-term cervical VNS, with the neuroanatomy of the brainstem nuclei involved in the mechanisms of action of VNS. Yellow: Dorsal Raphe Nucleus (DRN), red: Locus Coeruleus (LC), green: Nucleus Prepositus Hypoglossi (NPH), an inhibitory input to the LC and the Nucleus Paragigantocellularis (NPG), an excitatory input to the LC, blue: Nucleus Tractus Solitarius (NTS). **(A)** A helicoidal electrode is wrapped around the vagus nerve and stimulates the nerve. **(B)** The vagus nerve is composed of three types of fibers: **(A)** (large-diameter myelinated), **(B)** (small-diameter myelinated) and **(C)** (unmyelinated) fibers that have different electrical conductances. **(C)** An intensity-dependent increase in NE concentration following VNS was observed in the hippocampus of healthy rats for a range of intensities of 0.5–1 mA (20 Hz, 500 μs pulse width, and 30 s duration) (Roosevelt et al., 2006). Another study observed an increased concentration of extracellular NE in the hippocampus following VNS administration (1 mA, 30 Hz, 250 μs pulse width, 7 s ON, and 18 s OFF), with an increase of at least 70% preventing the development of pilocarpine-induced limbic seizures (Raedt et al., 2011). Blocking the α_2_-adrenoreceptor in the hippocampus of rats that were responders to VNS reversed the seizure-suppressing effects (Raedt et al., 2011). An increased activation of post-synaptic α_2_-adrenoreceptors located in a subfield of the Cornu Ammonis (CA3) of the hippocampus was observed after long-term VNS (2 weeks, 0.25 mA, 20 Hz, and 500 μs pulse width) (Manta et al., 2013). A study showed that VNS (1 mA, 20 Hz, and 500 μs pulse width) produced a persistent enhanced synaptic transmission between the perforant path and the CA3 region that was abolished when the LC was lesioned and when a β-adrenergic receptor antagonist was injected in the lateral ventricle (Shen et al., 2012). **(D)** During stimulation, a sigmoid-like relationship was found between the pupil dilation and the charge per pulse (Mridha et al., 2021). An increased pupil diameter was observed with an increasing current intensity and duration in a dose-dependent manner (Collins et al., 2021). **(E)** A significant increased NE was reached in the cortex following VNS with an intensity of 1 mA (20 Hz, 500 μs pulse width, and 30 s duration) (Roosevelt et al., 2006). Another study observed an increased level of extracellular NE in the prefrontal cortex after long-term VNS (2 weeks, 0.25 mA, 20 Hz, and 500 μs pulse width) (Manta et al., 2013). **(F)** A study using GCaMP6s imaging in Thy1-GCaMP6s mice revealed an increased fluorescence in NE axons within the dorsal cerebral cortex during VNS, with larger and longer lasting effects for higher intensities and longer pulse width stimulations (Collins et al., 2021). **(G)** An increase in ΔFosB was observed in the DRN after chronic VNS (3 weeks), but no increase in c-Fos was observed after acute VNS (2 h) in the DRN (0.25 mA, 20 Hz, 250 μs pulse width, 30 s ON, and 5 min OFF) (Cunningham et al., 2008). **(H)** An increased basal firing rate of the serotonergic neurons in the DRN was observed after long-term (14-, 21-, and 90-day) VNS treatments, but not after short-term (1-h, 1-day, and 3-day) VNS treatments (0.25 mA, 20 Hz, 500 μs pulse width, 30 s ON, and 5 min OFF) (Dorr and Debonnel, 2006). Another study showed an increased basal firing rate of the serotonergic neurons in the DRN after 14 days, but the increase was abolished when the LC was lesioned (0.25 mA, 20 Hz, 500 μs pulse width, 30 s ON, and 5 min OFF) (Manta et al., 2009). **(I)** An increased discharge rate of the LC was observed with acute VNS (0.3 mA, 20 Hz, and 500 μs pulse width) (Groves et al., 2005). An increased firing rate of the LC was found after short-term (1-h, 1-day, and 3-day) and long-term (14-, 21-, and 90-day) VNS treatments (0.25 mA, 20 Hz, 500 μs pulse width, 30 s ON, and 5 min OFF) (Dorr and Debonnel, 2006). An increased percentage of NE neurons displaying bursts and an increased number of bursts per minutes were observed in VNS-implanted rats after 14 days of VNS compared to control rats and the number of spikes per burst was even further increased after 90 days of VNS (0.25 mA, 20 Hz, 500 μs pulse width, 30 s ON, and 5 min OFF) (Manta et al., 2009). **(J)** A study showed that increasing the current intensity resulted in a shorter latency to the onset of a significant driven activity of the LC. Moreover, a monotonical increase of the neural activity in the LC was observed with increasing the current intensity as well as increasing the pulse width. Finally, higher-frequency stimulation yielded to a strong but transient activation of the LC, while lower-frequency trains lead to a smaller but longer activation of the LC (Hulsey et al., 2017). **(K)** The presence of the Arc protein was found in the LC after 10 min of VNS (1 mA, 20 Hz, 500 μs pulse width) (Shen et al., 2012). **(L)** A study observed ΔFosB in the LC after chronic VNS (3 weeks) (0.25 mA, 20 Hz, 250 μs pulse width, 30 s ON, and 5 min OFF) (Cunningham et al., 2008). **(M)** A regional induction of c-Fos in the LC was found after 3 h of VNS (1 mA, 30 Hz, 500 μs pulse width, 30 s ON and 5 min OFF) (Naritoku et al., 1995). Another study observed c-Fos in the LC after acute VNS (2 h) (0.25 mA, 20 Hz, 250 μs pulse width, 30 s ON, and 5 min OFF) (Cunningham et al., 2008). **(N)** Arc proteins were observed in the NTS after 10 min of VNS (1 mA, 20 Hz, and 500 μs pulse width) (Shen et al., 2012). *The scientific figure was partially created using the illustration Toolkit – Neuroscience from Motifolio, Inc.*

Originating from the cerebral cortex, the main feedback influences are shown from the orbitofrontal and anterior cingulate cortex (Aston-Jones and Cohen, 2005). Despite the vast efferent network of the LC, an injection study using tract-tracers revealed that mainly two areas constituted the major LC inputs: the nucleus paragigantocellularis, which is part of the ventrolateral rostral medulla and the nucleus prepositus hypoglossi, in the dorsomedial rostral medulla (Aston-Jones et al., 1991). However, other minor inputs to the LC have been identified in the dorsal cap of the paraventricular nucleus of the hypothalamus, the lateral parabrachial nucleus, the spinal lamina X, the preoptic area dorsal to the supraoptic nucleus, the Kölliker-Fuse nucleus and the mesencephalic reticular formation (Aston-Jones et al., 1991; Luppi et al., 1995). For subcortical structures, one of the few known ascending pathways are those from the NTS relaying vagal-related information. The relay between the NTS and the LC is conducted through two disynaptic pathways: an excitatory pathway (via the nucleus paragigantocellularis) and an inhibitory pathway (via the nucleus prepositus hypoglossi) (Ennis and Aston-Jones, 1988, 1989; Luppi et al., 1995; Ruffoli et al., 2011). The existence of these pathways was demonstrated through anatomical experiments in rats using retrograde tracers unilaterally injected in the LC (Aston-Jones et al., 1986). It was shown that in rats, electrical stimulation of the nucleus paragigantocellularis resulted in a phasic and excitatory response in the LC (Ennis and Aston-Jones, 1988), while electrical stimulation of the nucleus prepositus hypoglossi yielded to the inhibition of spontaneous discharge of LC neurons (Ennis and Aston-Jones, 1989). Additionally, in another electrophysiological study in rats, stimulation of the LC resulted in an antidromic stimulation of the neurons in the nucleus paragigantocellularis and the nucleus prepositus hypoglossi (Aston-Jones et al., 1991). The neuroanatomy of the noradrenergic pathway and the brainstem nuclei involved in the mechanisms of action of VNS are represented in Figure 1.

It is worth mentioning that other stimulation techniques have been investigated and may activate pathways that are similar to VNS. For example, trigeminal nerve stimulation has shown to reduce pentylenetetrazol-induced seizures in rats and seizure frequency in humans (Fanselow et al., 2000; DeGiorgio et al., 2003). The antiepileptic effects observed with trigeminal nerve stimulation could be linked to the existing connections of the trigeminal mesencephalic nucleus with the NTS, the LC or the lateral parabrachial nucleus (Luo et al., 1991; Allen et al., 1996; DeGiorgio et al., 2003; Schrader et al., 2011). Likewise, a study conducted in rats observed anticonvulsants (or proconvulsant effects, depending on the frequency of stimulation) with spinal cord stimulation (Jiao et al., 2016). In the field of neuropathic pain, a study conducted with rat-spared nerve injury models observed an increased discharge rate of the LC with spinal cord stimulation in responders to the therapy (Song et al., 2013). Although generalization of these results to epilepsy is not straightforward, the increased activity of the LC observed with spinal cord stimulation in a cohort of rats included in the study could suggest a common mechanism of action with VNS.

## Animal Vagus Nerve Stimulation Studies

Based on the widespread connections of the noradrenergic system and on the neurochemical properties of NE, VNS was hypothesized to reduce seizure development and trigger plastic changes within the brain, with the LC-NE system standing as the cornerstone of these effects. We will first review the body of evidence from animal studies, pointing at a critical involvement of NE in both short and long-term antiepileptic effects of VNS. A summary of the results of previous animal studies presented in this section can be found in Figure 1.

### Direct Involvement of the Locus Coeruleus

Krahl et al. (1998) conducted the first study that identified the LC as a key structure involved in the antiepileptic effects of VNS. In this study, the LC of rats were chemically lesioned with bilateral microinfusion of 6-Hydroxydopamine hydrobromide (6-OHDA), leading to a significant and chronic NE depletion in cortical areas and the hippocampus, as confirmed *ex vivo* with histological examination of regional brain-dissected samples. This study used the maximal electroshock seizure (MES) model, which is categorized as a model of generalized tonic-clonic seizures that elicits hindlimb extension (HLE) and often used to test anticonvulsant compounds (Castel-Branco et al., 2009). Using a transcorneal electrode, the MES was generated with a 60 Hz, 125 mA-alternating current delivered for 200 ms. Under active VNS (0.8 mA, 20 Hz, and 500 μs pulse width), researchers showed that rats with a lesioned LC showed increased seizure severity compared to animals with an intact LC (Krahl et al., 1998). Similar results were observed when the LC of rats were acutely inactivated by bilateral microinfusion of 1 μL 5% lidocaine hydrochloride into the LC for 2 min (Krahl et al., 1998). A histological verification of the injection was conducted after the MES test and only rats whose cannulas were located within 1 mm of the LC were included in the analyses. Therefore, it was suggested that the LC was involved in the antiepileptic effects of VNS by releasing NE that reduces seizure susceptibility and severity. This first evidence promoted the research to further characterize the firing of the LC during acute VNS.

Likewise, another study in rats measured the activity of the LC with implanted intracerebral electrodes and revealed a direct modulation of the LC activity during VNS (Groves et al., 2005). Indeed, an increased discharge rate of the LC was observed with acute VNS (0.3 mA, 20 Hz, and 500 μs pulse width) at an intensity known to maximally recruit A-fibers and some B-fibers in rats, but not C-fibers (Groves et al., 2005). The discharge rate of the LC neurons was determined over three post-stimulation epochs of 30 s and was compared to the baseline activity. A statistically significant increase of the firing rate was observed over the third epoch after VNS (Groves et al., 2005). A similar study exploring firing rates of NE neurons in the LC of healthy rats after short-term (1-h, 1-day, and 3-day) and long-term (14-, 21-, and 90-day) VNS treatments, revealed an increased firing rate after short-term and long-term VNS (0.25 mA, 20 Hz, 500 μs pulse width, 30 s ON, and 5 min OFF) (Dorr and Debonnel, 2006). Finally, a study characterized the LC firing in implanted rats after 14 and 90 days of VNS (0.25 mA, 20 Hz, 500 μs pulse width, 30 s ON, and 5 min OFF). Compared to control rats that were implanted with a dummy stimulator, the researchers found an increased percentage of NE neurons displaying burst activity, in addition to an increased number of bursts per minute in VNS-implanted rats after 14 days of VNS. The number of spikes per burst was even further increased after 90 days of VNS. It was hypothesized that this increased number of bursts per minute could be explained by a shift in the firing pattern of NE neurons toward burst discharging, therefore releasing a higher level of NE (Manta et al., 2009).

Further research supported the evidence of the VNS-activation of the LC and NTS. Ten minutes of VNS was shown to increase the presence of the activity-regulated cytoskeleton (Arc) protein, a marker of neural activity, as a result of increased gene transcription in those nuclei following VNS (1 mA, 20 Hz, and 500 μs pulse width) (Shen et al., 2012). In addition, a regional induction of c-Fos, a nuclear protein expressed in highly activated neurons, was also observed in the LC following 3 h of VNS administration (1 mA, 30 Hz, 500 μs pulse width, 30 s ON, and 5 min OFF), while no expression of c-Fos immunoreactivity was observed in rats with a sham stimulation (Naritoku et al., 1995). Finally, an immunochemistry study probed the presence of short- and long-term markers of neuronal activation (c-Fos and ΔFosB, respectively), in rats that received acute (2 h) or chronic (3 weeks) VNS (0.25 mA, 20 Hz, 250 μs pulse width, and 30 s ON every 5 min). Acute VNS significantly increased c-Fos in the LC and chronic VNS lead to an increased ΔFosB in the LC (Cunningham et al., 2008).

### Locus Coeruleus-Dependent Modulation of the Dorsal Raphe Nucleus

Neurochemical techniques that used tracers highlighted reciprocal connections between the LC and the Dorsal Raphe Nucleus (DRN), a major serotonin brainstem nucleus (Luppi et al., 1995; Kim et al., 2004). Serotonin depletion in the brain was shown to lower the threshold of audiogenically- (i.e., triggered by an acoustic stimulation), chemically- and electrically-evoked seizures in rats (Bagdy et al., 2007). Compounds that increase the extracellular level of serotonin can inhibit focal (limbic) seizures and generalized seizures (Bagdy et al., 2007). It is therefore likely that the LC and the DRN interact during VNS to confer the antiepileptic effects.

The basal firing rate of the LC and the DRN were recorded after short-term and long-term VNS (0.25 mA, 20 Hz, 500 μs pulse width, 30 s ON, and 5 min OFF) in healthy rats (Dorr and Debonnel, 2006). Although the LC showed an increased firing rate after both short-term and long-term VNS, the firing rate of the DRN only showed a significant increase after long-term VNS. It was therefore hypothesized that the LC mediates the activity of serotonergic neurons through α_1_-adrenoreceptors. Indeed, by increasing the norepinephrine tone on α_1_-adrenoreceptors located on the cell body of serotonergic neurons, the LC can tonically activate serotonergic neurons in the DRN (Baraban and Aghajanian, 1980). This was the first evidence suggesting that while VNS activates NE neurons first, long-term stimulation can modulate the activation of other types of neurons such as the serotonergic neurons of the DRN. The dependence of serotonergic DRN neurons on the NE modulation in VNS is further supported by a study that showed a significant increase of DRN-located serotonergic basal firing rate after 14 days of VNS (0.25 mA, 20 Hz, 500 μs pulse width, 30 s ON, and 5 min OFF), which was not observed when NE neurons in the LC were priorly lesioned with a selective noradrenergic toxin DSP-4 (Manta et al., 2009).

Finally, while Cunningham et al. (2008) showed an increase in c-Fos production in the LC of rats after acute VNS (2 h), no increase in c-Fos could be observed in the DRN after short-term VNS. However, a significant increase in ΔFosB was observed in both the LC and DRN after chronic VNS (3 weeks). Therefore, the improved seizure control over time (Elliott et al., 2011) could be explained by a progressive modulation of the DRN neuronal activity by the LC.

### Noradrenergic Modulation of the Cerebral Cortex: Hippocampal Effects

In addition to the DRN, other studies showed that VNS increased the extracellular concentration of NE in several cortical areas (Roosevelt et al., 2006; Follesa et al., 2007; Manta et al., 2009) and in the hippocampus (Roosevelt et al., 2006; Raedt et al., 2011; Manta et al., 2013), which is part of the limbic system and often typically involved in the most common forms of temporal lobe epilepsy. Interestingly, an intensity-dependent increase in NE concentration following VNS was observed in the hippocampus of healthy rats (Roosevelt et al., 2006). While an intensity of 0.5–1 mA showed a significant increase of NE concentration in the hippocampus, a significant increase was reached in the cortex following a VNS current intensity of 1 mA only (500 μs pulse width, 20 Hz, and 30 s duration). The increased NE concentration was transient and returned to baseline after the stimulation periods ended (Roosevelt et al., 2006). Moreover, a study that used epifluorescence to image neuronal activity in NE neurons in Thy1-GCaMP6s mice, revealed a strong increase in fluorescence in NE axons within the dorsal cerebral cortex during VNS for different stimulation parameters (0.1, 0.4, and 0.8 mA; 30 Hz; 100, 500, and 800 μs applied for 5, 1, and 0.5 s), with larger and longer-lasting effects for higher intensities and longer pulse width stimulations (Collins et al., 2021).

A study characterizing the role of NE under VNS was further conducted in rats with limbic seizures evoked by intrahippocampal perfusion of the muscarinic agonist pilocarpine *via* a microdialysis probe (Raedt et al., 2011). This study revealed an increased concentration of extracellular NE in the hippocampus during VNS (1 mA, 30 Hz, 250 μs pulse width, 7 s ON, and 18 s OFF). Results indicated that an increased hippocampal NE concentration of at least 70% prevented the development of pilocarpine-induced limbic seizures. Therefore, it is reasonable to assume that a non-negligible increase in NE release (compared to the baseline NE activity) is crucial for observing antiepileptic effects with VNS. Considering that the increase of extracellular NE following VNS administration was not sufficient to prevent limbic seizures in all animals, the question of responsiveness to VNS arises. One could hypothesize that a genetic and environmental inter-individual variability lead to differences in functional or structural integrity of the noradrenergic network, therefore impacting the susceptibility of VNS to modulate the NE release to prevent seizure development. Moreover, this study emphasized the role of hippocampal NE in the antiepileptic effects delivered by VNS by administering a selective α_2_-adrenoreceptor antagonist in proximity of the seizure focus. Blocking the α_2_-adrenoreceptor in the hippocampus of rats that were responders to VNS reversed the seizure-suppressing effects (Raedt et al., 2011). Another study used microelectrodes to assess the activation of post-synaptic α_2_- and α_1_-adrenoreceptors in the hippocampus, as well as the level of extracellular NE in cortical areas after long-term VNS (2 weeks, 0.25 mA, 20 Hz, 500 μs pulse width, 30 s ON, and 5 min OFF) (Manta et al., 2013). An increased level of extracellular NE was observed in the prefrontal cortex and to a lesser extent, in the hippocampus. The results also showed an increased degree of activation of post-synaptic α_2_-adrenoreceptors located in a subfield of the Cornu Ammonis (CA3) of the hippocampus following VNS administration, while the degree of activation of α_1_-adrenoreceptors remained unaltered.

Finally, an electrophysiological study conducted in healthy rats showed that injection of NE in the pyramidal neurons in the CA3 reduced the firing of hippocampal neurons through the activation of the post-synaptic α_2_-adrenoreceptors (Curet and de Montigny, 1988). Together, these findings indicate that the antiepileptic effects of VNS are linked to the release of NE that influences the activation of α_2_-adrenoreceptors reducing the excitability of the hippocampus and therefore decreasing seizure susceptibility.

### Dose-Dependency of Locus Coeruleus Activation

Evaluating the level of activation of structures involved in the antiepileptic effects of VNS for different stimulation parameters is crucial to develop individual stimulation strategies that maximize the effects of VNS. In this context, while the implication of the LC in the antiepileptic effects of VNS appears as established (Dorr and Debonnel, 2006; Manta et al., 2009). Hulsey et al. (2017) investigated the neural activity in the LC in response to VNS for different current amplitudes, pulse frequencies, train durations, inter-train intervals and pulse widths. Increasing the current intensity and keeping the other stimulation parameters constant, led to a proportional significant phasic increase in the activity of the LC, in both single-unit and multi-unit recordings, over the entire investigated intensity range (0.1–2.5 mA). Moreover, latency of LC firing rate peak was independent of the stimulation intensity, compatible with the neural transmission delay from the vagal trunk to the LC. Longer trains of stimulations elicited, however, longer-lasting phasic responses, with return to baseline firing levels occurring only after the VNS offset. Since current intensity of 0.1 mA led to the activation of the LC, it seems that the activation of the LC resulted from the stimulation of A and B-fibers, while C-fibers were not necessary for LC activation (Hulsey et al., 2017). Interestingly, a plateau effect tendency was detected in a dog model for intensities beyond 1.2 mA, which corresponds to the intensities saturating A-fibers and B-fibers (Castoro et al., 2011). However, translation of this observed effect to humans requires caution. There may be a close mirroring between A/B-fibers and LC dose-dependency characteristics. One cannot exclude, nevertheless, that the slight increase in LC activity reported with current intensities over the saturation threshold of A and B fibers (Hulsey et al., 2017), results from the onset recruitment of C-fibers (McAllen et al., 2018).

In addition to varying the intensity, pulse widths were varied from 30 μs to 500 μs while keeping the current intensity constant, at 0.8 mA (Hulsey et al., 2017). As observed for the increasing intensity, a significant increase of neural activity in the LC was observed with an increasing pulse width. Complementary to the intensity and the pulse width, a linear relationship between charge per pulse and LC activity was observed up to 160 nC, where a plateau was then observed.

Finally, in addition to the previous parameters, the stimulation frequency was increased from 7.5 Hz to 120 Hz while other parameters were kept constant. Higher-frequency stimulation yielded to a strong but transient activation of the LC while lower-frequency trains led to a smaller but longer activation of the LC. Critically, however, histologic investigations showed that using a stimulation frequency of 50 Hz and above can cause irreversible damages to the vagus nerve with the development of endoneurial edema followed by early axonal degeneration of the large myelinated fibers (Agnew et al., 1989).

Taking advantage of the fact that variation in pupil diameter is a proxy to both phasic and tonic LC activity (Aston-Jones and Cohen, 2005), studies were conducted in rodents to investigate the influence of different VNS parameters on pupil size (Collins et al., 2021; Mridha et al., 2021). An increased pupil diameter was observed with an increasing current intensity and duration in a dose-dependent manner, with a trend more pronounced when the arousal state of the animals was lower before the stimulation (Collins et al., 2021). A sigmoid-like relationship was found between pupil dilation and the charge per pulse (Mridha et al., 2021), displaying a tendency toward saturability of pupil dilation at similar intensity ranges (0.7–0.9 mA), as shown from LC-recordings by Hulsey et al. (2017) However, this study did not provide a statistical demonstration of a threshold above which no further pupil dilation occurs. A steeper increase in the pupil response was observed when a higher frequency of stimulation was used (20 Hz > 10 Hz > 5 Hz) (Mridha et al., 2021). These results show that the frequency of stimulation may induce cumulative effects, leading to a higher vagal activation when a specific charge per pulse is used. It may be therefore important to consider frequency when interpreting VNS experiment results. Moreover, the frequency could be an important feature for the titration of stimulation parameters. Although other neurotransmission systems are at stake with respect to pupil size modulation (particularly the cholinergic transmission), this finding warrants further exploration of parametric VNS-induced pupil dilatations in humans, in whom direct neuronal recordings of the LC are not possible.

Based on the results summarized in this section, it appears that different stimulation parameters could lead to different patterns of NE release with different functional consequences (Hulsey et al., 2017). This may be linked to the two disynaptic pathways that exist between the NTS and the LC (Ennis and Aston-Jones, 1988, 1989; Luppi et al., 1995; Ruffoli et al., 2011). Indeed, the activation of the NTS with different stimulation paradigms could involve these pathways differently. Maximizing the activation of the excitatory compared to the inhibitory pathway could optimize the activation of the LC and therefore the subsequent release of NE. Moreover, due to inter-subject variability, different patterns of LC activation may be seen even though the same stimulation parameters are used.

### Effects on Neural Plasticity

In addition to the dose-dependent response to stimulation, studies also supported long-term neuroplastic effects of VNS. One study investigated how VNS could affect synaptic transmission in the hippocampus and evaluated the implication of the LC and β-adrenergic receptors (Shen et al., 2012). A recording electrode was placed into the CA3 region, and a stimulation electrode was placed at the perforant path. The electric field of excitatory post-synaptic potentials evoked by a single pulse stimulation of the perforant path were measured in CA3 before and after 10 min of VNS (1 mA, 20 Hz, and 500 μs pulse width). The results revealed a persistent enhanced synaptic transmission between the perforant path and the CA3 region after VNS administration. The effects were reversed if the LC was electrolytically lesioned and if a β-adrenergic receptor antagonist was injected in the lateral ventricle. These results further emphasize the role of the LC and β-adrenergic receptors in the plasticity triggered by VNS.

Other studies hypothesized that the release of NE in cortical areas and the hippocampus could lead to neuronal plasticity and neurogenesis that may cause long-term seizure suppressing effects (Roosevelt et al., 2006; Revesz et al., 2008). VNS was reported to increase the proliferation of progenitors in the hippocampus of adult rats (0.75 mA, 20 Hz, 250 μs pulse width, 30 s ON, and 5 min OFF) (Revesz et al., 2008). Moreover, VNS increased the extracellular concentration of NE in the hippocampus (1 mA, 30 Hz, 250 μs pulse width, 7 s ON, and 18 s OFF) (Raedt et al., 2011), while a higher level of NE was associated with a higher proliferation of neural progenitors in the hippocampus (Malberg et al., 2000; Kulkarni et al., 2008). Therefore, the increased proliferation of progenitors in the hippocampus following VNS administration may be mediated by hippocampal NE activity (Revesz et al., 2008). Hence, it is tempting to suggest that VNS acts *via* the LC-NE system to trigger molecular mechanisms in the hippocampus, leading to a tissue reorganization that restores proper cell behaviors and reduces seizure susceptibility.

## Evidence in Humans

**FIGURE 2:**
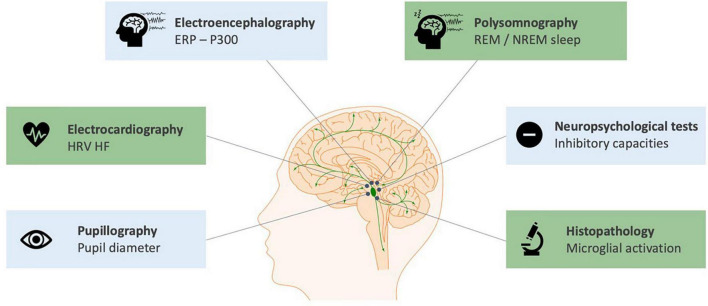
Biomarkers of the short-term (blue) and long-term (green) modulation of the LC-NE system with cervical VNS in humans. *The scientific figure was partially created using the illustration Toolkit – Neuroscience from Motifolio, Inc.*

In humans, the number of studies exploring the noradrenergic system in the context of VNS is more limited. This is due to: (i) the difficulty in obtaining direct neuroimaging, electrophysiological or histopathological findings on LC status; (ii) the complexity – involving a lumbar puncture – and non-established reliability of NE assessment from the cerebrospinal fluid (Laxer et al., 1979); (iii) the confounding role of concomitant medications in VNS patients. Nevertheless, several studies were conducted, mostly through neurophysiological measurements that reflected the activation of the LC-NE system and its modulation by VNS. An overview of the biomarkers of short-term and long-term modulation of the LC-NE system with VNS in humans can be found in Figure 2 and a complete summary of the results of studies presented in this section can be found in Supplementary Table 1.

### Event-Related Potential: P300

The effect of VNS on NE signaling was evaluated with the P300 component of event-related potentials during an oddball task paradigm (Hammond et al., 1992; Brázdil et al., 2001; De Taeye et al., 2014). The P300, i.e., a positive EEG deflection reflecting an underlying depolarization around 300 ms post-stimulus typically over central or parietal midline derivations, can be divided in two sub-components: an early P3a, more pronounced over the anterior regions, and a later P3b, rather detected over centro-posterior areas. The amplitude of the P300, and of the P3b in particular, is considered to reflect indirectly the phasic activity of the LC-NE system (Nieuwenhuis et al., 2005). De Taeye et al. (2014) suggested that the P300 amplitude could be used as a non-invasive biomarker for the therapeutic efficacy of VNS: a significant increase in the P300 amplitude was observed during a VNS ON condition compared to a VNS OFF condition in responders only. P300 amplitude could therefore constitute an interesting functional biomarker of the difference between responders and non-responders to VNS. However, two other studies did not observe a modulation of the P300 amplitude with VNS (Hammond et al., 1992; Brázdil et al., 2001). This negative finding may be explained by the small number of subjects included (≤10 patients) or because responders were not differentiated from non-responders (De Taeye et al., 2014).

Another study further evaluated the potential of P300 features recorded during stimulation to correctly classify responders and non-responders (Wostyn et al., 2017). In contrast to the study of De Taeye et al. that focused on the parietal midline Pz electrode (De Taeye et al., 2014), measurements from 60 electrodes were incorporated (Wostyn et al., 2017). Analyses indicated that the P300 amplitude was significantly increased not only in the Pz channel, but also in the right centro-parietal region in responders to VNS (Wostyn et al., 2017). A classification model was built with a cross-validation technique consisting in building a model based on a subset of patients (training set) and testing it on an independent data subset (validation set). While simply using the P300 amplitude measured by the Pz electrode led to a classification accuracy of 61%, summing the P300 amplitude measured by the CP2 electrode (right centro-parietal region) during the “VNS OFF” condition with the P300 amplitude measured by the PO5 channel (left parieto-occipital region) during the “VNS ON” condition led to a classification accuracy of 94%. However, further research is needed to determine whether the signals measured from the non-midline electrodes arise from the P300 wave or from other signals that could differ between responders and non-responders. Recently, a prospective study was conducted to measure the P3b amplitude of patients before the initiation of their treatment and after 1 year of VNS treatment (Hödl et al., 2020). The P3b was observed by means of a three-stimuli oddball task, including not only a rare target and a frequent distractor stimulus, but also a rare non-target stimulus (Nieuwenhuis et al., 2005). Compared to non-responders, patients who became VNS-responsive showed a significantly lower baseline P3b amplitude in the VNS OFF condition during the oddball task (Hödl et al., 2020). A lower baseline P3b amplitude has been associated with a lower baseline activity of the LC-NE system, potentially implying that responders to VNS present a lower baseline NE reactivity compared to non-responders (Hödl et al., 2020), allowing a greater relative increase of NE following VNS treatment.

### Pupil Diameter

As in animals, pupil diameter is tightly associated with both phasic and tonic activity of the LC (Joshi et al., 2016). A functional magnetic resonance imaging study indicated that the pupil diameter was positively correlated to the activity of the LC at rest and during an oddball task (Murphy et al., 2014). Based on these observations, a study was conducted to evaluate how VNS may affect pupil diameter in patients with refractory epilepsy at rest and during the light reflex condition (i.e., when light stimuli were presented to evaluate whether the pupil light reflex was modified when VNS was administered) (Desbeaumes-Jodoin et al., 2015). A significant increase in resting pupil diameter was observed in the VNS ON condition compared to the VNS OFF condition, suggesting the influence of acute VNS toward an increased tonic LC activity. However, the time course of the pupil response compared to stimulus onset was not evaluated in the study, and this precludes from establishing whether a VNS-evoked phasic response exists or not. Furthermore, no correlation with clinical response was found, nor any significant difference in the reactivity of the pupil during the light reflex conditions, between VNS ON and VNS OFF conditions (Desbeaumes-Jodoin et al., 2015).

### Neuropsychological Markers: Response Inhibition

In addition to the P300 event-related potential and the pupil diameter, the effects of VNS in response inhibition were also studied. Although the neurobiological processes involved in response inhibition are not totally elucidated yet, it was suggested that the noradrenergic system has a critical role. Indeed, the phasic activation of the LC-NE system facilitates responses to task-relevant processes (Aston-Jones and Cohen, 2005) and is therefore expected to be involved in response inhibition (Chmielewski et al., 2016). Moreover, it was shown that acute systemic administration of atomoxetine, a NE reuptake inhibitor that rapidly increases concentration of NE in the prefrontal cortex, leads to an increased response inhibition in rats and humans (Chamberlain et al., 2006; Robinson et al., 2008).

One study determined how VNS may influence response inhibition in a stop signal task, with the time needed for the epileptic patient to inhibit a response measured in both VNS ON and OFF conditions, in order to compute the inhibition gain (Schevernels et al., 2016). VNS decreased the time needed to inhibit a response and this effect was correlated to the clinical benefit of VNS. In addition, the P300 was also measured, as this may also reflect the response inhibition (Bekker et al., 2004; Lansbergen et al., 2007; Wessel and Aron, 2015). P300 amplitudes during response inhibition were larger during VNS but were independent of the clinical efficacy of VNS. Therefore, it was hypothesized that even if VNS does not reduce seizure frequency in all patients, it could still influence neurobiological processes such as NE transmission, without reaching clinical benefits (Schevernels et al., 2016).

Concerning inhibitory capacities, another study used the Eriksen Flanker test in epileptic patients with stimulation ON and OFF to measure their ability to suppress irrelevant information (van Bochove et al., 2018). In the Eriksen Flanker test, participants must assess the congruency of a central target (usually arrows or letters) according to their surrounding distractors. In line with previous results (Schevernels et al., 2016), this study showed an increased ability to suppress irrelevant information during stimulation in VNS-responders only. It is worth noting that the initial reaction time of responders in the VNS OFF condition was higher than non-responders. This slow reaction time in responders could be interpreted as a reduced NE reactivity when no stimulation is administered, that is restored in the VNS ON condition (van Bochove et al., 2018). These results bring new evidence of a differential modulation of the norepinephrine system with VNS between individuals.

### Sleep Physiology

When investigating biomarkers of the effects of VNS on the LC-NE system, research has also focused on sleep characteristics. As part of the ascending activating system, the LC promotes wakefulness, and it needs to be partly silenced for sleep initiation. Further silencing is required to go from slow wave sleep to Rapid-Eye Movement (REM) during which LC neurons are fully silent (Aston-Jones and Bloom, 1981). Increases of noradrenergic signaling in the brain, such as those induced by antidepressants and mono-oxidase inhibitors, were proved to reduce REM-sleep prevalence and to lead toward more non-REM sleep and wakefulness (Siegel, 2011). A study investigated sleep parameters of patients with refractory epilepsy at baseline and after chronic VNS to determine whether VNS induces changes in their sleep structures (Rizzo et al., 2003). After chronic VNS, a decreased REM-sleep in implanted patients was reported (Rizzo et al., 2003). In addition, it was shown that VNS shortened overall nocturnal sleep duration with an increased wakefulness during both the night and the day, with no apparent impact on daytime sleepiness (Rizzo et al., 2003). Furthermore, a reduced number of (REM) sleep episodes and duration were observed in patients with refractory epilepsy who are implanted with a VNS device (Rizzo et al., 2003). Therefore, one could hypothesize that VNS increases the activity of NE neurons in the LC, which leads to a reduction of REM-sleep and promotes wakefulness in implanted patients. However, it should be pointed out that many factors can also influence REM sleep (e.g., medications, co-morbid depression, sleep habits, etc.). For example, some patients in this study received pharmacologic polytherapy including carbamazepine, an antiepileptic drug that has been associated with a decreased REM sleep (Yang et al., 1989; Drake et al., 1990; Placidi et al., 2000). In children with refractory epilepsy, another study reported a significant increase in deep slow wave sleep (non-REM 3 sleep stage) and non-REM 1 sleep stage (in percentage and duration), as well as a reduced sleep latency after 9 months of VNS compared to baseline (Hallböök et al., 2005). Finally, in a prospective study conducted by Hödl et al. (2020), responders to VNS showed significantly higher non-REM 3 sleep compared to non-responders, prior to the initiation of their treatment. These results could reflect variability in the LC-NE system of patients with refractory epilepsy.

### Heart Rate Variability

In order to find biomarkers that could refine the selection of responders to VNS, a prospective study also measured the heart rate variability (HRV) at baseline and after 1 year of VNS treatment (Hödl et al., 2020). The high frequency power of the HRV (HRV HF) reflects the parasympathetic activity and is calculated based on the fluctuation of the time between adjacent heartbeats (Malik et al., 1996; Shaffer and Ginsberg, 2017). VNS responders showed significantly lower HRV HF compared to non-responders at baseline and after 1 year of VNS treatment. While a lower HRV HF might be associated with a higher sympathetic LC-related activity, it was suggested that a more complex network connectivity pattern recruiting other structures of the vagal afferent network could be involved (Hödl et al., 2020). However, these results also suggest that a variation in the vagal afferent network exists between individuals, which could relate to the different responsiveness to VNS in patients with refractory epilepsy.

A study used machine learning to build prediction models for evaluating responsiveness to VNS therapy preoperatively based on HRV indices in wake and sleep states (Fang et al., 2021). 52 HRV indices were evaluated in 30 responders and 29 non-responders before the implantation of a VNS device. After feature selection, the best outcome prediction was observed when assessing HRV indices during sleep compared to the wake state. Indeed, the model built using a univariate filtering method for feature selection of sleep indices reached an accuracy of 74.6% while the best model in the wake state reached an accuracy of 68.8%. The HRV HF index was respectively, ranked 4th best predictor index in the sleep state, and 3rd best predictor index in the wake state.

### Histopathology

While immunochemistry studies evaluating the effects of VNS on the vagal afferent network in humans are scarce due to the limited number of samples available, a first study was recently conducted with aim to extract morphological characteristics of the nuclei linked to this network after chronic VNS (Ding et al., 2021). Neuronal cell number, astrogliosis and microglial activation in the LC, the NTS and the rostral pontine group of raphe nuclei were assessed in 4 autopsy cases with history of refractory epilepsy treated with VNS and 4 chronic epilepsy cases without VNS treatment. A trend toward a decreased microglial activation was observed in the left LC (stimulated side) compared to the right LC in VNS cases. A similar trend was observed between the left LC of VNS cases and the left LC of non-VNS cases. Results were not statistically significant but could suggest a slightly decreased neuroinflammation in the LC with long-term VNS. No difference in the neuronal cells number and the number of astrocytes was observed in these nuclei. Further studies using other labels of neuronal activation and neurotransmitter concentrations could further characterize the plastic effects of long-term VNS (Ding et al., 2021).

### Transcutaneous Vagus Nerve Stimulation

Besides the clinically approved cervical VNS, an emerging approach to stimulate the vagus nerve non-invasively consists of the so-called transcutaneous VNS (tVNS). This non-invasive method uses a surface-electrode to stimulate the cymba concha (i.e., inner part of the auricle, innervated by the auricular branch of the vagus nerve) (Yap et al., 2020) and is believed to share common patterns of brain activation with cervical VNS (Frangos et al., 2015). Recent studies used psychophysiological and biological indices to provide evidence of the activation of the noradrenergic system with tVNS. Indeed, a functional magnetic resonance imaging study showed that stimulation of the cymba concha resulted in the activation of regions of the vagal afferent network (e.g., ipsilateral NTS, bilateral spinal trigeminal nucleus, DRN, LC, contralateral parabrachial area, amygdala, and nucleus accumbens) compared to a sham stimulation applied on the earlobe (Frangos et al., 2015). A randomized, double-blind controlled trial was conducted to assess the efficacy and safety of tVNS (Bauer et al., 2016). A significant reduction in seizure frequency was observed in the tVNS group (*n* = 39, 25 Hz, 0.5 ± 0.47 mA, 250 μs pulse width, 30 s ON, 30 s OFF) after 20 weeks of tVNS therapy, that was not observed in the control group (*n* = 37, 1 Hz, 1.02 ± 0.83 mA, 250 μs pulse width, 30 s ON, and 30 s OFF), while the responder rates were similar between the two groups. One recent study reported a robust tVNS-evoked pupillary dilation in healthy volunteers that was not observed under sham stimulation on the earlobe (Sharon et al., 2020). In contrast, other studies did not report differences in pupil size between the tVNS ON and tVNS OFF conditions at rest and during an oddball task (Keute et al., 2019; Warren et al., 2019). Discrepancies may be partially explained by the different methodologies adopted or the different stimulation paradigms used. Indeed, Sharon et al. (2020) observed a pupil dilation during a task-free resting condition with short tVNS pulses (duration of 3.2 s) and a variable, subject-dependent maximal current intensity below pain threshold (mean intensity of 2.2 ± 0.24 mA). By contrast, the two studies that failed to observe an increased pupil diameter during stimulation were using longer 30-s stimulations in healthy subjects, during an oddball task with a stimulation of 3 mA (25 Hz, 200 μs pulse width) (Keute et al., 2019) or 0.5 mA (25 Hz, 200–300 μs pulse width) (Warren et al., 2019) that was constant across subjects. These findings may suggest a higher impact of tVNS on the phasic LC activation (evoked by short stimulation trains), rather than on the modulation of its tonic activity, which would have been mirrored in mean pupil size differences between tVNS ON and tVNS OFF conditions, and preferentially induced by longer tVNS trains.

Another study measured the level of NE released with tVNS by the means of hormonal indices such as the salivary alpha amylase and the salivary cortisol (Warren et al., 2019). Salivary alpha amylase is a digestive enzyme known to be associated with the activity of the LC-NE system (Warren et al., 2019). The presence of this enzyme as well as the presence of salivary cortisol, an arousal-related stress hormone, were suggested to be markers of the central NE activity (Hill et al., 2003; Ehlert et al., 2006; Warren et al., 2017). Interestingly, an increased salivary alpha amylase was observed when tVNS was used during an oddball task (Warren et al., 2019), which did not occur when a sham stimulation to the earlobe was used. Finally, in the sham condition, salivary cortisol seemed to decrease with time during the set of stimuli-discrimination tasks. It was suggested that this overall decrease in cortisol secretion could be the reflection of mental fatigue of the participants, the activities carried out during the experiments and other factors (Warren et al., 2019). However, when tVNS was used, this tendency of decreased salivary cortisol was abolished.

Further research is needed to establish a classification model that intends to predict responsiveness to cervical VNS, based on non-invasive markers of the activity of the noradrenergic system when tVNS is delivered. If such a model reaches a high accuracy, tVNS could be an exciting avenue for assessing responsiveness to invasive VNS before the implantation of a neurostimulator and therefore identify more selectively VNS responders to avoid unnecessary surgery and possibly related complications.

## Discussion and Conclusion

The results presented in this review indicate the important implication of NE in the antiepileptic effects of VNS. Based on the results presented, one could hypothesize that responders to VNS show a lower NE reactivity prior to the implantation of the device compared to non-responders (Hödl et al., 2020), allowing a greater relative increase of NE release with VNS. This difference in reactivity may partially be explained by some variability in the NE receptors or in the efficacy of the NE reuptake transporter across individuals (Hödl et al., 2020). Therefore, it is suggested that inter-individual differences in the functional integrity of the LC-NE system exist and may, at least partially, explain the variability in responsiveness to VNS.

Although clear evidence of VNS-induced dose-dependent LC activation is present in animals, more solid findings should still be sought in humans. Particularly, a promising opportunity might reside in the use of LC-NE system markers (such as pupil diameter or P300 responses) as parametric read-outs of the afferent effects of VNS, and studies aiming at a clearer definition of their dose-dependent characteristics are thus advocated. Potential differences in the phasic or tonic LC modulation, depending on the cervical or transcutaneous nature of the vagal stimulation, should also be explored.

In addition to the aforementioned biomarkers, which indirectly reflect the activity of the LC-NE system, further studies could evaluate the structural and functional properties of the LC itself with imaging techniques and determine whether differences exist between responders and non-responders to VNS. Magnetic resonance imaging studies might be useful to extract structural and functional properties of structures of the vagal afferent network, which could be critically involved in the antiepileptic effects of VNS. Recently, techniques have been developed to evaluate the integrity of the LC *in vivo* (Sasaki et al., 2006; Matsuura et al., 2013; Miyoshi et al., 2013; Ohtsuka et al., 2013; Chen et al., 2014; Watanabe et al., 2014; Takahashi et al., 2015; Betts et al., 2017, 2019; Priovoulos et al., 2018; Dahl et al., 2019; Liu et al., 2019; Guinea-Izquierdo et al., 2021), using neuromelanin-sensitive magnetic resonance imaging (Keren et al., 2015). Neuromelanin is a metabolite of neuronal NE synthesis that accumulates inside noradrenergic neurons of the LC (Fedorow et al., 2006). These imaging techniques of the LC have been used in the context of Alzheimer’s disease (Miyoshi et al., 2013; Takahashi et al., 2015; Betts et al., 2019), Parkinson’s disease (Sasaki et al., 2006; Matsuura et al., 2013; Miyoshi et al., 2013; Ohtsuka et al., 2013), schizophrenia (Watanabe et al., 2014) and late-life major depressive disorder (Guinea-Izquierdo et al., 2021), but never in the context of refractory epilepsy. Comparing structural and functional properties of the LC *in vivo* between responders and non-responders could help to develop new biomarkers of responsiveness to VNS. Using predictive analytic models (such as classification models of the integrity and the modulation of the LC-NE system based on a combination of multiple biomarkers), could help to refine the selection of patients before the implantation of the device and improve the current understanding of the mechanisms of action of VNS.

Further research leading to the personalization of the stimulation strategy to maximize the antiepileptic effects of VNS and potentially increase the response rate to the therapy is warranted. One option could be to investigate how to preferentially activate the excitatory pathway that links the NTS to the LC *via* the nucleus paragigantocellularis, as well as minimize the activation of the inhibitory pathway that relays the information through the nucleus prepositus hypoglossi. Functional magnetic resonance imaging could again be a useful tool to develop a stimulation paradigm based on individualized titration. Indeed, it could be used to assess the activation of the LC during stimulation under different stimulation parameters. However, research about the acute effects of VNS using functional magnetic resonance imaging has been limited due to technical considerations and the requirements specified by the constructor of the VNS device (LivaNova, 2019). The development of fully MRI-compatible VNS devices will allow to stimulate during MRI acquisitions and might lead to the possibility of determining individual stimulation parameters leading to the highest LC activation, with the best functional consequences as part of a personalized stimulation-based therapy.

## Author Contributions

AB, SV, and RE contributed to the analysis and interpretation of the relevant literature and drafting of the manuscript. All authors with their complementary backgrounds have critically revised the manuscript, making a substantial, direct, and intellectual contribution to the work. All authors approved it for publication.

## Conflict of Interest

AB and PD were employed by the company Synergia Medical SA. The remaining authors declare that the research was conducted in the absence of any commercial or financial relationships that could be construed as a potential conflict of interest. The authors declare that this study received funding from Synergia Medical and the Walloon Region. The funder was not involved in the analysis and the interpretation of the literature, the writing of this article or the decision to submit it for publication.

## Publisher’s Note

All claims expressed in this article are solely those of the authors and do not necessarily represent those of their affiliated organizations, or those of the publisher, the editors and the reviewers. Any product that may be evaluated in this article, or claim that may be made by its manufacturer, is not guaranteed or endorsed by the publisher.
