# Supplementary material for: How Is the Norepinephrine System Involved in the Antiepileptic Effects of Vagus Nerve Stimulation?
Source: Front Neurosci. 2021 Dec 2;15:790943. doi: 10.3389/fnins.2021.790943 (PMC8675889; doi:10.3389/fnins.2021.790943)
Supplement: Supplementary file 1 [file Data_Sheet_1.pdf]

Supplementary Material

1 Supplementary Table

TABLE 1: Studies in humans using biomarkers to evaluate the activation of the noradrenergic system with (t)VNS.

TABLE 1: Studies in humans using biomarkers to evaluate the activation of the noradrenergic system with (t)VNS.

Author	Year	Number of subjects included	Type of VNS	Stimulation parameters	LC-NE related outcome measured	Results
E.J. Hammond et al.	1992	9 patients with DRE	Cervical VNS	The effects of the stimulation amplitude, duration and rate were studied: these parameters were varied.<br><br>- Current: 0.0-3.5 mA<br>- Frequency: 1-50 Hz<br>- Pulse width: 130-1000 μs	Effect of VNS on **auditory evoked potentials (ERPs).**	No effect on auditory-ERPs was observed during VNS and after 1 year of VNS treatment.
M. Brádzil et al.	2001	10 patients with DRE	Cervical VNS	Patient-specific parameters in the ranges:<br><br>- Current: 0.5-1 mA<br>- Frequency: 30 Hz<br>- Pulse width: 500 μs	**Auditory and visual event-related potentials** during an oddball task were recorded at two-time points: before the implantation of a VNS device and 3-6 months after the activation of the device.	No significant difference was found in the latency or amplitude of the auditory ERPs. A significant increase in the amplitude of the visual P3 potential was observed, with a maximal increase in the frontal region (Fz electrode).

P. Rizzo et al.	2003	10 patients with DRE (9 completed the study)	Cervical VNS	Patient-specific parameters in the ranges:<br><br>- Current: 1.25 -3.25 mA<br>- Frequency: 30 Hz<br>- Pulse width: 250-500 μs	Polysomnography was recorded in DRE patients before the implantation of the device and after chronic VNS. The following parameters were evaluated: **sleep and REM latency, wakefulness after sleep onset, total sleep time, sleep efficiency, total duration of REM sleep (minutes and %), duration (minutes and %) of each REM stage (1,2,3 and 4), awakenings, arousals and stage shifts.** Sleep-wake diary data allowed to evaluate the sleep quantity (total sleep time and daytime naps), sleep quality (easy awakening), daytime alertness	An increased number of awakenings and an increased wakefulness after chronic VNS was observed. Moreover, sleep stage 1 increased and REM sleep decreased (total duration, percentages, and number of episodes). Overall, the data collected proposed a shortened nocturnal sleep with increased wakefulness during the night and the day without decreased alertness during the daytime. Patients reported an easier awakening and did not perceive their sleep as less restorative. Sleep onset was not

					and wake quality (attention, mood and quality of life).	delayed and the number of stage shifts and arousals were not affected by VNS.
T. Hallböök et al.	2005	15 children with DRE	Cervical VNS	Patient-specific parameters in the ranges:<br><br>- Current: 1-1.5 mA<br>- Frequency: 30 Hz<br>- Pulse width: 500 μs<br><br>(The current was initially set at 0.25 mA at the end of the surgical procedure and was increased in steps of 0.25 mA during the initial 4 weeks, up to a current intensity of 1-1.5 mA.)	Patients were examined with ambulatory polysomnographic recordings at baseline and after 3 and 9 months of VNS treatment. **Sleep latency, sleep stages (percentage and duration), movement time and delta power** were recorded.	A longer nocturnal sleep, an increased slow wave sleep (NREM 3 sleep stage) and NREM 1 sleep stage were observed in VNS-implanted children after 9 months of VNS-treatment. Moreover, a significant decrease is sleep latency was observed after 9 months of treatment.
L. De Taeye et al.	2014	20 patients with DRE: 10 responders to VNS and 10 non-responders to VNS	Cervical VNS	Patient-specific parameters in the ranges:<br><br>- Current: 0.75-3 mA<br>- Frequency: 20-30 Hz<br>- Pulse width: 250-500 μs	The **P3 amplitude** was measured in the VNS ON condition and the VNS OFF condition during an oddball paradigm and was compared between responders and	A significant increase in the P3 amplitude was observed during the VNS ON condition compared to the VNS OFF condition in responders only.

					non-responders to VNS.	
E. Frangos et al.	2015	12 healthy subjects	Transcutaneous VNS	Two sessions were realized for each subject:<br><br>*<u>tVNS condition</u>:*<br>- Current: 0.3-0.8 mA<br>- Frequency: 25 Hz<br>- Pulse width: 250 μs<br>- Applied in the left cymba conchea region<br><br>*<u>Sham condition</u>*:<br>- Current: 0.3-0.9 mA<br>- Frequency: 25 Hz<br>- Pulse width: 200-300 μs<br>- Applied on the left earlobe	The **BOLD response** (fMRI) was evaluated to determine whether tVNS applied on the cymba conchea can activate the NTS and the "classical" vagal projections. Brain regions that responded to tVNS were compared to regions that responded to the activation of the earlobe.	Stimulation of the cymba conchae resulted in the activation of regions of the vagal afferent network (e.g. ipsilateral NTS, bilateral spinal trigeminal nucleus, dorsal raphe, locus coeruleus, contralateral parabrachial area, amygdala and nucleus accumbens) compared to the sham stimulation.
V. Desbeaumes-Jodoin et al.	2015	34 subjects: 22 with DRE and 12 with refractory depression. 5 patients with refractory depression and 8 patients with DRE did not meet the inclusion criteria. Therefore, the study included 21 patients:	Cervical VNS	Patient-specific parameters in the ranges:<br><br>- Current: 0.25-2.25 mA<br>- Frequency: 20-30 Hz<br>- Pulse width: 250-500 μs	**Resting pupil size and light reflex measures** (when light stimuli were presented) were compared in consecutive intervals in VNS ON and VNS OFF conditions.	In the VNS ON condition, a significant increased resting pupil diameter was observed but no difference in the light reflex was observed. No difference in the pupil diameter nor light reflex was

		14 with DRE and 7 with major depression				observed between DRE patients and patients with major depression.
H. Schevernels et al.	2016	20 patients with DRE: 10 responders to VNS and 10 non-responders to VNS	Cervical VNS	Patient-specific parameters in the ranges:<br><br>- Current: 0.75-3 mA<br>- Frequency: 20-30 Hz<br>- Pulse width: 250-500 μs	Patients performed a stop-signal task in VNS ON and VNS OFF conditions and the **inhibitory performance** in those two conditions was evaluated (based on the stop-signal reaction time, or SSRT, an index of the time needed to inhibit a response). **The event-related potentials and the pupil size** were also measured.	Patients who clinically benefit from VNS treatment also show a faster response inhibition when the vagus nerve is stimulated (SSRT difference in the VNS ON and VNS OFF condition correlated with the percentage of seizure reduction).<br><br>Larger P3 amplitudes were observed when the vagus nerve was stimulated but was independent of the clinical efficacy of the therapy.<br><br>The pupil was more dilated in stop signals but was not

						significantly affected by VNS.
S. Wostyn et al.	2017	20 patients with DRE: 10 responders to VNS and 10 non-responders to VNS	Cervical VNS	Patient-specific parameters in the ranges:<br><br>- Current: 0.75-3 mA<br>- Frequency: 20-30 Hz<br>- Pulse width: 250-500 μs	VNS was switched ON and OFF and the EEG responses of responders were compared to the EEG responses of non-responders during an auditory oddball task. EEG recordings (60 channels, with the Extended International 10-10 System) were used to evaluate the **features of the P3 wave (peak latency and peak amplitude)** in those two conditions. P3 amplitudes were extracted from all EEG channels at the moment of the peak amplitude in the Pz reference channel. A classification	No difference in the P3 latency was found between the responders and non-responders to VNS and between the VNS ON and VNS OFF conditions.<br><br>The P3 amplitude was significantly increased in the channels Pz and CP2 in responders only. Channels C4 and C6 showed a significant decrease in P3 in non-responders only.<br><br>A classification model based on the amplitude of the P3 wave as measured in the Pz reference electrode can correctly classify

					model of responsiveness was built with the cross-validation technique based on the EEG features.	responders and non-responders with an accuracy of 61%. Although it is not clear whether results from the non-midline electrode originated from the P3 wave or other brain-dependent signals, a classification model based on the P3 amplitude measured in the CP2 channel in the VNS OFF condition and the PO5 channel in the VNS ON condition led to a classification accuracy of 94%.
M.E. van Bochove et al.	2018	19 patients with DRE (10 responders and 9 non-responders, but two patients, one responder and one non-responder, did not complete the study due to fatigue)	Cervical VNS	Patient-specific parameters in the ranges:<br><br>- Current: 0.75-3 mA<br>- Frequency: 20-30 Hz<br>- Pulse width: 250-500 μs	Patients executed the Eriksen flanker task during the VNS ON condition and the VNS OFF condition and the **distractor interference** was	A significant modulation of the general reaction time was observed for responders during stimulation. The congruency effect is reduced in responders with

					indexed by the congruency effect as measured by the difference in reaction time between the congruent and incongruent trials.	stimulation and responders were substantially faster during stimulation than in the VNS OFF condition. A reduced reaction time with stimulation was not observed in non-responders. Without VNS, responders have a relatively slow reaction time compared to non-responders but the general reaction time of responders is comparable to non-responders in the VNS ON condition.
C. Warren et al.	2019	Experiment 1a: 24 healthy subjects<br><br>Experiment 1b: 20 healthy subjects<br><br>Experiment 2: 17 healthy subjects	Transcutaneous VNS	Two sessions were realized for each subject:<br><br>*<u>tVNS condition</u>:*<br>- Current: 0.5 mA<br>- Frequency: 25 Hz<br>- Pulse width: 200-300 µs<br>- Applied in the cymba conchea region	Experiment 1a: subjects performed "classical oddball" and "novelty oddball" tasks in the visual and auditory modalities and	<u>P3 amplitude</u><br>Experiment 1a: tVNS did not significantly affect oddball P3 amplitude nor novelty P3 amplitude.

				<u>Sham condition</u>:<br>- Current: 0.5 mA<br>- Frequency: 25 Hz<br>- Pulse width: 200-300 μs<br>- Applied on the left earlobe	the **amplitude of the P3 wave** was recorded. Saliva samples were collected at different time points to evaluate the **secretion of salivary alpha amylase and salivary cortisol**.<br><br>Experiment 1b: a simplified version of the oddball task was conducted with a higher number of trials compared to experiment 1a. **The amplitude of the P3** was measured but no saliva sampled were collected.<br><br>Experiment 2: the participants realized a cued task switching task. The **pupil size** was measured at	Experiment 1b: the oddball P3 was larger in the tVNS condition than in the sham condition, but the difference was not significant.<br><br><u>Pupil size</u><br>Experiment 2: tVNS did not significantly affect pupil size.<br><br>*<u>Saliva samples:</u>*<br>An analysis of the pooled data from saliva samples (experiment 1a and experiment 2) indicated that tVNS increased the salivary alpha amylase secretion compared to the baseline, that was not observed in the sham condition. Moreover, tVNS worked against a general tendency for salivary cortisol secretion to

					different time points and the **secretion of salivary alpha amylase and salivary cortisol** was measured, but the amplitude of the P3 was not recorded.	decrease over the course of a session.
M. Keute et al.	2019	33 healthy subjects	Transcutaneous VNS	Two sessions were conducted for each subject :<br><br>*tVNS condition:*<br>- Current: 3 mA<br>- Frequency: 25 Hz<br>- Pulse width: 200 μs<br>- Applied in the left cymba conchea region<br><br>*Sham condition*:<br>- Current: 3 mA<br>- Frequency: 25 Hz<br>- Pulse width: 200 μs<br>- Applied on the left earlobe	Subjects performed an auditory oddball task and the **resting pupil size, pupillary responses and reaction time** to target stimuli were measured.	In the resting measurement, no significant difference in the mean overall pupil size was observed between the sham and the tVNS conditions.<br><br>In the auditory oddball task, no significant difference was observed between the sham condition and the tVNS condition. Moreover, reaction time to target stimuli did not significantly differ

						between the two conditions.
S. Hödl et al.	2020	30 patients with DRE	Cervical VNS	Patient-specific parameters in the ranges:<br><br>- Current: 0.75-2.5 mA<br>- Frequency: 30 Hz<br>- Pulse width: 500 μs	**Polysomnography (% of NREM 1, NREM 2, NREM 3 and REM sleep in total sleep time)**, **heart rate variability (HRV) and cognitive-related potentials (P3b)** were recorded before the initiation of the therapy and after 1 year of VNS treatment and were compared between responders and non-responders.	15 out of 30 patients who were implanted became responders to VNS. Three neurophysiological parameters were found to be significantly different in patients who became responders to VNS prior to the initiation of their treatment. Indeed, before the initiation of the treatment, the NREM3 sleep stage was significantly higher, the amplitude of the P3b (in the VNS OFF condition) was significantly lower and the HF (high frequency) HRV was significantly lower in patients with DRE who

						became responders to VNS.
O. Sharon et al.	2020	25 healthy subjects (one excluded due to excessive blinking)	Transcutaneous VNS	Two sessions were conducted for each subject:<br><br>*tVNS condition:*<br>- Current: 2.2 ± 0.24 mA<br>- Frequency: 25 Hz<br>- Pulse width: 200-300 μs<br>- Applied in the cymba conchea region<br><br>*Sham condition*:<br>- Current: 2.79 ± 0.27 mA<br>- Frequency: 25 Hz<br>- Pulse width: 200-300 μs<br>- Applied on the earlobe	High-density EEG and pupillometry were used to measure the **alpha oscillations and the pupil size**, during visual fixation at rest when short trials (3.4s) of transcutaneous VNS and sham stimulation (i.e. somatosensory stimulation of the earlobe) were used.	Transcutaneous VNS leads to a robust dilation of the pupil compared to the sham stimulation. A greater attenuation of alpha oscillations was observed with tVNS compared to the sham stimulation.
J.J. Ding et al.	2021	4 autopsy cases with history of DRE treated with VNS (VNS+)<br><br>4 age- and sex matched chronic epilepsy cases without VNS (VNS-)	Cervical VNS	VNS+ group: patient-specific parameters in the ranges:<br><br>- Current: 1.2-3.5 mA<br>- Frequency: 30 Hz (not reported for one case)<br>- Pulse width: 250-500 μs (not reported for one case)	With immunochemistry techniques, **the number of neuronal cells, astrogliosis and microglial activation in brainstem nuclei along the vagal afferent pathway (LC, NTS and rostral pontine**	No statistically significant results were found, but a trend towards a decreased microglial activation was observed in the left LC (stimulated side) compared to the right LC in the VNS+ cases. A same trend was

					**group of the raphe nuclei)** were investigated and compared between VNS+ and VNS- cases.	observed between the left LC of VNS+ cases and the left LC of VNS- cases.
X. Fang et al.	2021	50 healthy controls and 59 patients with DRE: 30 became responders and 29 were non-responders	Cervical VNS	HRV features evaluated preoperatively in patients with DRE and healthy controls.<br><br>Patient-specific parameters in the ranges:<br><br>Responders:<br>- Current: 1.4±0.6 mA<br>- Frequency: 29.5±1.5 Hz<br>- Pulse width: 441.7±105.7 μs<br><br>Non-responders:<br>- Current: 1.5±0.4 mA<br>- Frequency: 28.8±3.9 Hz<br>- Pulse width: 431±111.7 μs	**Time domain, frequency domain, and non-linear indices of HRV** were compared between 30 responders and 29 non-responders in wake and sleep states.	49 HRV indices showed significant differences between DRE patients and healthy controls. Machine learning was used to build a model for evaluating responsiveness to VNS therapy prior the implantation of the device. The best outcome prediction was observed when assessing HRV indices during sleep compared to the wake state. The model reached an accuracy of 74.6% with sleep state indices, while the model based on wake indices

						reached an accuracy of 68.8%. The HRV HF index was ranked 4th best predictor index in the sleep state and 3rd best predictor index in the wake state.
